# Circulating fibroblast growth factor-23 is associated with increased risk for metachronous colorectal adenoma

**DOI:** 10.4103/1477-3163.76723

**Published:** 2011-02-12

**Authors:** Elizabeth Jacobs, Maria Elena Martinez, Julie Buckmeier, Peter Lance, Melissa May, Peter Jurutka

**Affiliations:** Epidemiology and Biostatistics, Mel and Enid Zuckerman College of Public Health, Arizona Cancer Center, University of Arizona, P.O. Box 245024, Tucson, AZ 85724-5024, USA

**Keywords:** Adenoma, cancer, colorectal, fibroblast growth factor-23, fibroblast growth factor

## Abstract

**Background:**

Fibroblast growth factor-23 (FGF-23) is a phosphaturic peptide and a key component of an endocrine feedback loop along with the hormonal vitamin D metabolite 1,25(OH)_2_D. Vitamin D has been shown to be inversely related to colorectal neoplasia; therefore, we hypothesized that the effect of FGF-23 on vitamin D metabolite concentrations could have implications for the risk of colorectal neoplasia.

**Materials and Methods:**

The purpose of this study was to prospectively evaluate the association between circulating concentrations of FGF-23 and the risk of metachronous (recurrent) colorectal adenomas. FGF-23 levels were assessed in 100 male and female participants from the Ursodeoxycholic Acid Trial, 50 of whom had a metachronous colorectal adenoma and 50 who did not.

**Results:**

Compared to the lowest tertile of FGF-23, the adjusted odds ratios (95% CIs) for the second and third tertiles were 2.80 (0.94 to 8.31) and 3.41 (1.09 to 10.67), respectively (*P*-trend=.03). In a linear regression model, there was also a statistically significant inverse relationship between FGF-23 and 1,25(OH)_2_D (β-coefficient=–1.2; *P*=.001). In contrast, no statistically significant trend was observed between FGF-23 and 25(OH)D concentrations (β-coefficient=0.55; *P*=.10).

**Conclusions:**

The current work presents novel preliminary evidence of a relationship between FGF-23 and the risk for colorectal neoplasia. FGF-23 activity may be mediated through biologic effects on individual serum and colonic 1,25(OH)_2_D levels, or it may be independent from the vitamin D pathway. Further studies in larger populations are necessary for confirmation and expansion of these hypothesis-generating results.

## BACKGROUND

Fibroblast growth factor-23 (FGF-23) is a peptide hormone member of the FGF-19 subfamily, which is differentiated from the larger FGF family by virtue of its lacking the conventional FGF heparin-binding domain and by exhibiting endocrine function.[1] FGF-23 has a critical role in phosphate homeostasis,[1,2] and signaling is mediated via a complex formed by FGF-23, FGFR1c, and Klotho.[1] FGF-23 is a key component of an endocrine feedback loop along with the hormonal vitamin D metabolite 1,25(OH)_2_D such that it is both induced by and represses the synthesis of 1,25(OH)_2_D in a concerted effort to maintain both calcium and phosphate homeostasis.[3] The regulation of 1,25(OH)_2_D by FGF-23 could also have implications for cancer, given that vitamin D metabolites have been shown to have been shown to have anticarcinogenic effects.

The hormone 1,25(OH)_2_D has antiproliferative and pro-differentiating effects in cancer cell lines.[4] It inhibits growth in colorectal cancer cell lines.[5] Epidemiological investigations have consistently shown that low serum levels of 25(OH)D, the precursor to 1,25 (OH)_2_D, are significantly related to increased risk of both colorectal adenomas and cancer.[6] Given the antineoplastic effects of 1,25 (OH)_2_D, the regulation of this hormone by FGF-23 may be of importance in colorectal carcinogenesis.

While there have been a number of studies related to vitamin D and cancer endpoints, few have investigated the potential role of FGF-23 in this pathway. One study reported significantly increased concentrations of FGF-23 in patients with advanced ovarian cancer compared to those with early-stage cancer or controls.[7] However, to date, no epidemiological investigations of FGF-23 and risk for colorectal neoplasia have been reported. Because of the critical role of FGF-23 in the maintenance of 1,25 (OH)_2_D and the potential importance of the latter in colorectal carcinogenesis, the present study was performed to assess whether circulating concentrations of FGF-23 were associated with the risk of metachronous colorectal adenoma, the precursor to colorectal cancer.

## MATERIALS AND METHODS

Study subjects for the current work were participants in the Ursodeoxycholic Acid (UDCA) Trial conducted at the Arizona Cancer Center.[8] This double-blind, placebo-controlled study was conducted to determine the effect of UDCA on metachronous colorectal neoplasia.[8] Participants had a colonoscopy-detected adenomatous polyp resected prior to randomization in the trial. A total of 1192 participants completed the UDCA trial by having a follow-up colonoscopy after 3 years.[8] No effect of UDCA on metachronous neoplasia observed. A total of 100 participants who had previously-collected data for the vitamin D metabolites 25(OH)D and 1,25(OH)_2_D were selected for analysis of plasma FGF-23 concentrations. From among the UDCA Trial participants who had a metachronous adenoma found at follow-up colonoscopy and measured vitamin D metabolites, 50 were randomly selected for inclusion into our study, along with 50 who did not have a metachronous lesion. Blood samples for these analyses were collected at baseline and were stored at –80°C. This work was approved by the University of Arizona Human Subjects Committee and Institutional Review Board.

### Endpoint ascertainment

Metachronous colorectal neoplasia was defined as any adenoma or cancer detected by colonoscopy at least 6 months after randomization to the UDCA Trial. These lesions have previously been called adenoma recurrences, but the possibility exists that some of the apparent ‘recurrent’ lesions are those that were missed at baseline colonoscopy. Therefore, it was recommended that the terminology be changed from ‘recurrent’ to ‘metachronous.’ Endoscopy and pathology reports were reviewed at each study site, and data regarding size, histology, number, and location were extracted. Central pathology review was then conducted at each site.

### Analysis of FGF-23, 1,25(OH)_2_D, and 25(OH)D

Plasma FGF-23 concentrations were measured with an enzyme-linked immunosorbent assay (ELISA). The C-terminal ELISA was performed according to the manufacturer’s instructions (Immutopics International, San Clemente, CA). Plates were analyzed on a Synergy 2 Multi-Mode Microplate Reader using Gen5^™^ Reader Control and Data Analysis Software (Bio-Tek Instruments Inc., Winooski, VT), reading optical density at 450 nm with a correction wavelength of 620 nm. All samples had an absorbance value within the limits of detection of between 18 and 445 RU/ml. The high (mean value of 305.35±5.83 RU/ml) and low (mean value of 33.36±2.33 RU/ml) controls produced inter-assay CVs of 12.33% and 9.27%, respectively. The intra-assay coefficient of variance for FGF-23 was less than 9.0%. Measurement of 1,25(OH)_2_D and 25(OH)D was conducted at the laboratory of Dr Bruce Hollis at the Medical University of South Carolina using established methods.[9,10] The coefficient of variation for the 1,25(OH)_2_D assay was 11.5%, while for the 25(OH)D assay it was less than 7.0%.

### Statistical analyses

All analyses were conducted using the STATA statistical software package (version 9.0, Stata Corporation, College Station, TX). For comparisons of baseline characteristics by the presence or absence of metachronous lesions, *P*-values were calculated, with chi-square analyses for categorical variables and *t*-tests for continuous variables. Linear regression models and scatter plots were used to assess the association between FGF-23 concentrations and levels of the vitamin D metabolites 25(OH)D and 1,25(OH)_2_D. For determination of the association between FGF-23 and the odds of metachronous adenoma, unconditional logistic regression modeling was used. Potential confounding variables were assessed using logistic regression modeling and included age, sex, race, body mass index, family history of colorectal cancer, presence of polyps prior to baseline colonoscopy, use of aspirin, current smoking, and blood concentrations of the vitamin D metabolites 25(OH)D and 1,25(OH)_2_D. Confounders were defined as variables that changed the point estimate by 10% or greater; the final model thus included age, sex, family history of colorectal cancer (yes or no), and self-reported polyp prior to baseline colonoscopy (yes or no). Tests for trend were conducted using the final adjusted regression model and a categorical variable for circulating FGF-23 concentration.

## RESULTS

Baseline characteristics of study participants with and without a metachronous adenoma are presented in Table 1. Among these participants, those who had a metachronous adenoma were significantly older than those who did not (68.1 years *vs* 64.6. years, respectively). Those with a metachronous lesion were also were more likely to have reported a polyp prior to the qualifying colonscopy for study entry and were less likely to have reported a family history of colorectal cancer, though these results were not statistically significant.

**Table 1:** Baseline participant characteristics classified by presence or absence of a metachronous colorectal adenoma

Characteristic	Metachronous adenoma n=50	No metachronous adenoma n=50	*P*-value
Male, n (%)	26 (52.0)	33 (66.0)	0.16
Age (years; mean ± SD)	68.1 ± 7.0	64.6 ± 9.0	0.03
Body mass index (kg/m^2^; mean ± SD)	27.6 ± 4.5	27.4 ± 4.5	0.83
Family history of colon cancer* Yes, n (%)	11 (22.0)	19 (38.0)	0.08
Polyps prior to baseline colonoscopy† Yes, n (%)	25 (53.2)	16 (34.4)	0.06
Current smoking Yes, n (%)	6 (12.0)	6 (12.0)	1.00
Race white, n (%)	43 (86.0)	39 (78.0)	0.29

* History of colorectal cancer in one or more first-degree relatives,

† History of polyps prior to qualifying colorectal adenoma;

data are missing for six study participants.

**Figure 1:**
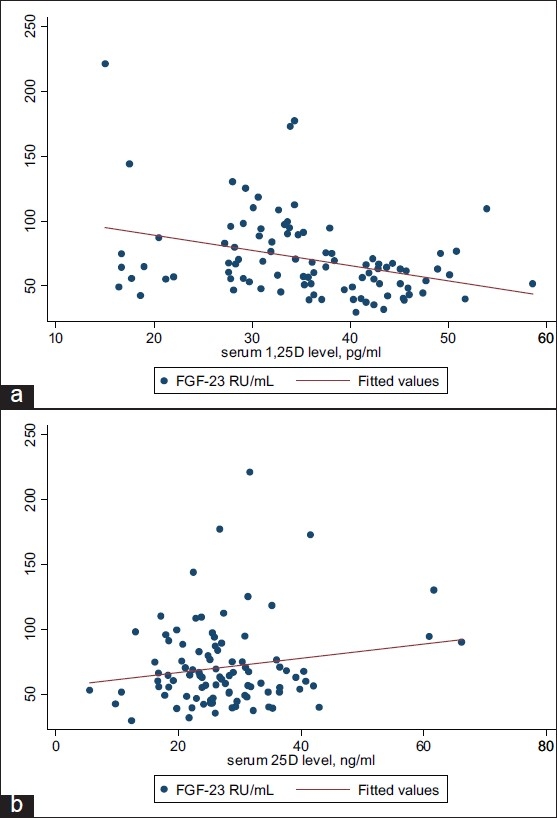
Plasma concentrations of FGF-23 by 1,25(OH)_2_D (panel a) and 25(OH)D (panel b) concentrations. Analyses were conducted using linear regression modeling with vitamin D metabolites as the continuous outcome variable and FGF-23 as the continuous exposure variable.

Figure 1 shows the association between FGF-23 and the vitamin D metabolites 1,25(OH)_2_D (panel A) and 25(OH)D (panel B), along with a fitted linear regression line. There was a statistically significant inverse relationship between FGF-23 and 1,25(OH)_2_D (β-coefficient=–1.2; *P*=.001), with the highest concentrations of FGF-23 being associated with the lowest levels of 1,25(OH)_2_D. Conversely, there was a suggestion of a direct association between FGF-23 and 25(OH)D concentrations, though this relationship did not reach statistical significance (β-coefficient=0.55; *P*=.10).

Table 2 shows the crude and adjusted odds ratios (ORs) and 95% confidence intervals (CIs) for metachronous adenoma by tertile of FGF-23 concentrations. Compared to the lowest tertile of FGF-23, the adjusted ORs (95% CIs) for the second and third tertiles were 2.80 (0.94 to 8.31) and 3.41 (1.09 to 10.67), respectively. The *P* for trend for the relationship between increasing FGF-23 concentration and odds for adenoma was statistically significant (*P*=.03).

**Table 2:** Odds of metachronous adenoma classified by tertile of FGF-23

Tertile of FGF-23 (RU/ml; mean±SD)	Metachronous adenoma/total	Crude OR (95% CI)	Adjusted OR* (95% CI)
1 (n=34) 44.8 ± 6.4	12/34	1.00	1.00
2 (n=33) 63.1 ± 5.5	18/33	2.20 (0.82 to 5.87)	2.80 (0.94 to 8.31)
3 (n=33) 104.1 ± 33.1	20/33	2.82 (1.05 to 7.60)	3.41 (1.09 to 10.67)
*P*-trend		0.04	0.03

* Odds ratio adjusted for age, sex, family history of colorectal cancer, and self-reported prior colorectal polyp.

## DISCUSSION

The current work presents novel preliminary hypothesis-generating evidence for a potential role for FGF-23 in colorectal neoplasia. A statistically significant inverse association between the phosphaturic hormone FGF-23 and odds for metachronous colorectal adenoma was observed. FGF-23 is a key regulator that suppresses the expression of kidney CYP27B1, the biosynthetic enzyme that catalyzes the production of circulating 1,25(OH)_2_D, the potent hormonal form of vitamin D.[1] This biological relationship was reflected in the findings of the present study, which showed that concentrations of FGF-23 and 1,25(OH)_2_D were significantly inversely associated. These results provide evidence that FGF-23 affects the amount of 1,25(OH)_2_D available for antineoplastic activity.

Both antiproliferative and pro-differentiating effects of 1,25(OH)_2_D on cancer cell lines have been reported,[4] along with growth inhibition in colorectal cancer cell lines.[5] Several mechanisms of action have been proposed to explain the inhibitory action of vitamin D on carcinogenesis. Induction of G_0_/G_1_cell-cycle arrest by 1,25(OH)_2_D in HL-60 human leukemia and breast cancer cell lines has been reported, possibly via increased expression of the cyclin-dependent kinase inhibitor proteins p27 ^Kip^.[4] Colorectal carcinoma cell differentiation may be induced by 1,25(OH)_2_D-mediated transcription of E-cadherin, a tumor suppressor gene; also, 1,25(OH)_2_D inhibits the expression of bcl-2, a suppressor of apoptosis, in HL-60 cells.[11] In addition, 1,25(OH)_2_D has been shown to reduce the transcriptional activity of b-catenin, a key proto-oncogene in colorectal carcinogenesis.[12–14] Given the potential for antineoplastic activity by 1,25(OH)_2_D, the influence of FGF-23 on the production of this hormone is a critical consideration.

As reviewed by Marsell[1] and Liu,[15] modulation of phosphate homeostasis and vitamin D metabolism in the kidney are the principal functions of FGF-23. The biological interaction between 1,25(OH)_2_D and FGF-23 is intricate and appears to manifest itself via phosphate regulation.[15] In the intestine, 1,25(OH)_2_D stimulates absorption of calcium and phosphate; 1,25(OH)_2_D and phosphate also induce expression and release of FGF-23, primarily from bone, to prevent phosphate levels from rising too rapidly.[2,15] In turn, FGF-23 inhibits further synthesis of 1,25(OH)_2_D by inducing the kidney vitamin D 24-hydroxylase (CYP24A1), which catalyzes 1,25(OH)_2_D catabolism,[2] as well as by suppressing the kidney CYP27B1. These functional effects are reflected in preliminary data from our current work, which show an inverse relationship between circulating 1,25(OH)_2_D and FGF-23 concentrations. We propose that the observed suppression of 1,25(OH)_2_D by FGF-23 results in an increased odds for metachronous adenoma in individuals with higher FGF-23 concentrations compared to those with lower levels.

One of the problems in research related to vitamin D and cancer is the epidemiological *vs* the laboratory approach to studying this relationship. In epidemiological work, 25(OH)D is most commonly employed as the vitamin D biomarker as it represents both endogenous synthesis and dietary/supplemental intake. However, 25(OH)D exhibits marked variation in humans due to factors such as degree of skin pigmentation, body size, physical activity, sun exposure, and diet.[6] In contrast, 1,25(OH)_2_D, the hormonal form of vitamin D, is more tightly regulated within the circulation and varies less markedly in human populations, but it is rarely studied in epidemiological work. The question therefore remains as to how higher concentrations of 25(OH)D can translate into the antiproliferative pro-differentiating effects of 1,25(OH)_2_D that are observed in molecular studies. One possible explanation is that the colonocyte possesses FGF-23 receptors[16] and also expresses the CYP27B1 enzyme. Variation in circulating levels of FGF-23 could potentially modulate intracellular production of colonocyte 1,25(OH)_2_D via CYP27B1, using serum 25(OH)D as the metabolic precursor. Thus, FGF-23 can not only modulate serum 1,25(OH)_2_D levels to regulate phosphate homeostasis, but the same hormone could impact local intracrine production of 1,25(OH)_2_D in the colon to elicit measureable differences in health outcomes.

Despite the relationship between FGF-23 and 1,25(OH)_2_D, the possibility remains that FGF-23 has independent effects on colorectal neoplasia or may act via another pathway, perhaps related to body size. Prior work has demonstrated that higher circulating FGF-23 is significantly associated with increased risk for cardiovascular disease, which shares many common risk factors with colorectal cancer, though the mechanism of action is currently unknown.[1] A direct relationship between FGF-23 and body weight has been reported;[17] however, in the present study no relationship between body mass index and FGF-23 was observed (data not shown). The reasons for these differences are unclear but merit further investigation with a larger sample size in order to better understand the epidemiology and biology of FGF-23.

The strengths of the current work include its prospective design and the fact that circulating concentrations of FGF-23, 1,25(OH)_2_D, and 25(OH)D were measured. However, some limitations of the study must be acknowledged. In order to ensure that FGF-23 concentrations were measured in those with data available for vitamin D metabolites, participants were not randomly selected from the original trial population overall, which may have introduced bias. Additionally, there was an imbalance in some of the baseline characteristics between those who had an adenoma recurrence and those who did not. Although we adjusted for potential confounders in the logistic regression models, the possibility of residual confounding remains. The greatest limitation of the work is that the sample size of 100 participants is small and does not allow for detailed examination of interactions between FGF-23, 1,25(OH)_2_D, or 25(OH)D and the outcome, and this will have to be addressed in future work. Therefore, this work should be considered preliminary and hypothesis-generating in nature.

## CONCLUSIONS

The present study provides the first evidence of an important and novel association between FGF-23 and the risk for colorectal neoplasia. This work supports a potential mechanism of action through the vitamin D pathway, though it is not clear whether FGF-23 acts via 1,25(OH)_2_D or has independent effects. A larger study population along with well-designed and complementary biochemical experiments will be essential for clarifying whether FGF-23 and vitamin D metabolites are acting independently or in concert to affect colorectal carcinogenesis.

## DECLARATION OF COMPETING INTERESTS

The author(s) declare that they have no competing interests.

## AUTHORS’ CONTRIBUTIONS

ETJ wrote the majority of the manuscript and contributed substantially to the concept, design, analyses, and interpretation of the data. MEM and PL assisted with the concept and design of the study as well as with manuscript preparation. JAB and MM made critical contributions to the acquisition of samples and the conduct of the FGF-23 assays. PWJ made major contributions to the concept, design, and interpretation of the data and also assisted with manuscript writing.

## AUTHORS’ PROFILE

**Dr. Maria Elena Martinez,** is a Professor of Epidemiology and Biostatistics at the Mel and Enid Zuckerman College of Public Health and the Arizona Cancer Center. She is the co-director of Cancer Prevention and Control at the Arizona Cancer Center.

**Dr. Peter Lance,** is co-director of Cancer Prevention and Control at the Arizona Cancer Center.

**Dr. Peter W. Jurutka,** is a molecular biologist with expertise in vitamin D biochemistry and molecular biology.

**Ms. Melissa R May,** is a research technician at the Arizona Cancer Center.

**Ms. Julie A Buckmeier,** is a research specialist at the Arizona Cancer Center.

**Dr. Elizabeth T Jacobs,** is a cancer epidemiologist at the Arizona Cancer Center with an interest in lifestyle and genetic risk factors for colorectal cancer. She currently has a primary interest in the vitamin D pathway and risk for cancer.
